# RAGE plays key role in diabetic retinopathy: a review

**DOI:** 10.1186/s12938-023-01194-9

**Published:** 2023-12-19

**Authors:** ZhiWen Lu, Bin Fan, YunZhi Li, YiXin Zhang

**Affiliations:** Department of Ophthalmology, The Second Hospital of Jilin University, Nanguan District, No. 4026, Yatai Street, Changchun, 130000 Jilin Province China

**Keywords:** RAGE, AGE, NF-κB, HMGB1, Diabetic retinopathy, Inflammation, Neovascularization, Treatment

## Abstract

RAGE is a multiligand receptor for the immunoglobulin superfamily of cell surface molecules and is expressed in Müller cells, vascular endothelial cells, nerve cells and RPE cells of the retina. Diabetic retinopathy (DR) is a multifactorial disease associated with retinal inflammation and vascular abnormalities and is the leading cause of vision loss or impairment in older or working-age adults worldwide. Therapies aimed at reducing the inflammatory response and unnecessary angiogenesis can help slow the progression of DR, which in turn can save patients’ vision. To maximize the efficacy and minimize the side effects, treatments that target key players in the pathophysiological process of DR need to be developed. The interaction between RAGE and its ligands is involved in a variety of cytopathological alterations in the retina, including secretion of inflammatory factors, regulation of angiogenesis, oxidative stress, structural and functional changes, and neurodegeneration. In this review, we will summarize the pathologic pathways mediated by RAGE and its ligand interactions and discuss its role in the progression of diabetic retinopathy to explore potential therapeutic targets that are effective and safe for DR.

## Introduction

Diabetic retinopathy (DR), a multifactorial disease primarily associated with retinal inflammation and vascular abnormalities, is the leading cause of vision loss or impairment in older and working-age adults worldwide [1]. By 2030, the number of patients affected by DR will rise to 191 million [2]. RAGE is a member of the immunoglobulin superfamily of cell surface molecules and was originally identified as a receptor that recognizes advanced glycation end products (AGEs). In addition to AGEs, there are other ligands that bind to RAGE, including members of the S100 family, HMGB-1, β-amyloid peptide, and Mac-1 [3]. As a component of the innate immune response, RAGE is expressed in many tissues, modulates a range of pathophysiological responses by activating associated signal transduction cascades [4], and induces the production of pro-inflammatory cytokines, growth factors, cell adhesion molecules, neurodegeneration, and ROS production. Pathological mechanisms involved in neurodegenerative diseases, cardiovascular diseases, lung diseases, chronic inflammation, diabetic retinopathy and cancer. In this article, we will summarize the role of pathologic pathways mediated by RAGE and its ligand interactions in the progression of diabetic retinopathy.

## Inflammatory factors play a pathogenic role in the progression of DR

DR is considered to be a chronic subclinical inflammatory disease, the most obvious link between inflammatory response and retinal dysfunction in DR involves retinal vascular damage and pathological neovascularization [5, 6]. The continuous production of inflammatory factors mediates the synthesis of acute proteins that initiate and maintain the inflammatory process in the vascular wall, resulting in retinal microvascular abnormalities and the breakdown of the blood-retinal barrier [7]. Retinal inflammation in DR promotes the production of vascular growth factors, cytokines and chemokines, inducing neovascularization and exacerbating the progress of DR [8]. Multiple clinical studies have demonstrated that patients with DR have increased levels of inflammatory markers in serum, vitreous and retina, including CRP, IL-6, TNF-α, NF-κB, and ICAM-1. This suggests that the inflammatory processes play a considerable role in the pathogenesis of DR, and inhibition of these inflammatory factors may slow or prevent the development of DR-specific lesions [5, 9, 10].

## Neovascularization is the characteristic manifestation of proliferative diabetic retinopathy

Retinal neovascularization is the characteristic pathological manifestation of proliferative DR (PDR). The neovascularization from the inner retinal vessels extends into the vitreous cavity. These immature, fragile and hemorrhagic pathologic neovascularization may induce hemorrhage and leakage, leading to vitreous hemorrhage (VH), further traction of retinal detachment (RD), and serious visual impairment. If not intervene, it will eventually develop to neovascular glaucoma or even blindness [2, 9, 11, 12]. Diabetic macular edema (DME) is caused by the accumulation of exudate due to disruption of the blood-retinal barrier (BRB), which can occur at any stage of DR and result in loss of vision in patients.

## Delay the progression of DR is necessary

Inflammation and angiogenic mediators lead to a stress response in retinal glial cells characterized by enhanced production of glial fibrillary acidic protein (GFAP), and impaired function or death of photoreceptor cells, which may ultimately lead to blindness [12, 13]. Therefore, DR leads to structural and functional abnormalities in retinal tissue as well as neurodegeneration, resulting in irreversible loss of vision. Therapeutic interventions are needed to delay or prevent the progression of the disease and to prevent complications. Therapeutic agents that exhibit anti-inflammatory and/or anti-angiogenic activity have been widely used to slow the progression of DR and treat complications, such as intravitreal injection of anti-VEGF inhibitors and steroids [9]. However, in view of the high price of anti-VEGF inhibitors and they often need to be used repeatedly, which is a great economic burden for both patients and society; patients tend to have poor compliance; and half of the patients do not respond to intravitreal injection of VEGF inhibitors [12], whereas steroid hormone therapy can increase the incidence of cataract and glaucoma. It is necessary to develop new pharmacological targets to inhibit DR progression at an early stage and prevent complications such as macular edema (ME), VH and RD from occurring, thereby saving patients' vision.

## RAGE and its ligands are involved in mediating pathological processes

### RAGE participates in mediating the production of inflammatory factors

RAGE is expressed in glial cells, Müller cells, nerve cells, vascular endothelial cells, pericytes and RPE cells, and can also be detected in retinal pathological response cells, including the preretinal membrane and the neovascular membrane [4]. RAGE is central to the pathogenesis of diabetes related complications and involves damage to multiple cell types. Multiple ligands interact with RAGE to activate NF-κB and increase VEGF release, resulting in intracellular oxidative stress and inflammation, which are participate in the pathogenesis of DR [14]. Anti-RAGE therapy can improve DR by reducing blood glucose levels, TNF-α levels and VEGF levels [15].

NF-κB, a protein complex that controls DNA transcription and produces a large number of cytokines, is an important regulator of many genes involved in inflammation, cell proliferation, apoptosis and immune responses. In the retina, NF-κB is confined to the subretinal membrane and microvessels and is activated in the early stage of DR. Activation of NF-κB is thought to be a key signaling pathway through which NF-κB binds to nuclear DNA and regulates the expression of several genes, leading to increased production of free radicals and ultimately inducing cell death. This explains why high glucose induces apoptosis of endothelial cells and pericytes in early DR lesions. Activated NF-κB also regulates the expression of genes encoding pro-inflammatory cytokines (IL-1β, IL-6, IL-8, TNF-α, VCAM-1 and ICAM-1) and chemokines, promoting retinal inflammatory response and oxidative stress, and further exacerbating the development of DR [9, 16, 17]. In the retina, activation of NF-κB enhances the expression of adhesion molecules that allow leukocytes to adhere to the surface of retinal vascular endothelial cells, a feature of inflammation and an early manifestation of pathological changes in DR. Leukocytes adhere to the endothelial wall via CD18 binding to ICAM-1 and VCAM-1, resulting in early BRB disruption, increased vascular permeability, endothelial cell damage and death, capillary nonperfusion, vascular occlusion, and neuronal loss of function and cell death [7, 18]. When anti-intercellular adhesion molecule-1 (ICAM-1) antibodies were administered to diabetic model animals, the pathological reactions associated with leukocytes were significantly reduced. ICAM-1 and CD18 mediated leukocyte adhesion is increased in the retinal vessels of diabetic model mice and explains many of the signature lesions of DR. Therefore, ICAM-1 and CD18 have been identified as potential therapeutic targets that, in combination with appropriate glycemic control, could be used to manage the vision-threatening complications of diabetes [19].

## RAGE-AGEs interactions activate intracellular signaling pathways

**Fig. 1:**
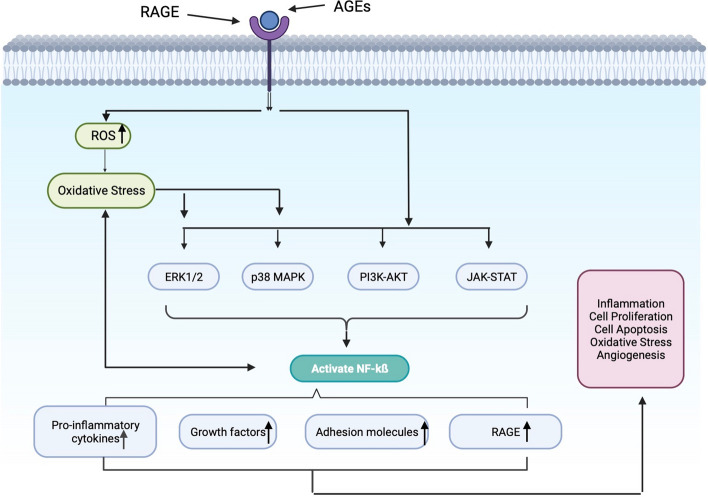
RAGE-AGEs interaction activates intracellular signaling pathways

AGEs are stable metabolic end products caused by slow non-enzymatic glycation (Maillard reaction) of ketones or aldehydes with proteins and lipids. AGEs accumulate during normal metabolism and aging, and their formation are accelerated under the action of inflammation, oxidative stress and hyperglycemia [20, 21]. AGEs-mediated damage occurs primarily through interaction with RAGE on cell membrane. Binding of RAGE and AGEs leads to increased oxidative stress and activation of various downstream signaling pathways, including Ras/ERK1/2, Ras/p-38MAPK, PI3K-AKT, Jak-STAT etc., and decreased activity of endothelial nitric oxide synthase (eNOS), which can directly or indirectly activate the NF-κB signaling pathway. Inducing increased expression of pro-inflammatory cytokines, growth factors, adhesion molecules and RAGE itself. This will lead to the secretion of inflammatory factors, cell proliferation, apoptosis, reactive oxygen species (ROS) generation, NO synthesis, angiogenesis and various vascular homeostasis functions imbalance etc., which have been demonstrated to play an important role in the progression of DR [22–24]. Limiting or avoiding the formation of AGEs and the combination of AGEs and RAGE can inhibit the progression of DR, making it a target for the prevention and treatment of DR and related complications (Fig. 1).

RAGE combines with AGEs to increase free radical-reactive oxygen species (ROS) production by activating nicotinamide adenine dinucleotide phosphate (NADPH) oxidase NoX to promote redox imbalance, leading to oxidative stress. ROS also activates MAPK signaling cascades, including ERK, p38MAPK and JNK. These kinases regulate the activity of downstream transcription factors (e.g., ATF-2, AP-1 and NF-κB), and trigger the up-regulation of a cascade of pro-inflammatory and growth factors by facilitating the nuclear translocation of NF-κB and the expression of TLR-2 and TLR-4. Up-regulation of vascular endothelial growth factor (VEGF), tumor necrosis factor (TNF-α), chemokines (CCL2, CCL5, CCL12), and adhesion molecules (ICAM-1 and VCAM-1) induces inflammation in the retina [2].

RAGE binding to AGEs directly induces mitogen-activated protein kinases (MAPKs) pathway, promotes the translocation of NF-κB, and upregulates pro-inflammatory cytokines, adhesion molecules and growth factors, leading to retinal cell dysfunction and neuroinflammation [11].

## The S100 protein family and Mac-1 bind to RAGE to promote inflammation

The S100 protein family interacts with RAGE and activates NF-κB, inducing the production of pro-inflammatory cytokines that lead to the translocation of neutrophils, monocytes and macrophages, inducing neuroinflammation and activation of glial cell. S100B is a neurotrophic factor that regulates cytoplasmic Ca^2+^ and cytoskeletal integrity in astrocytes, oligodendrocytes, neural progenitors, Langerhans' cells and dendritic cells [25]. When secreted extracellularly, S100B acts as a potent pro-inflammatory factor that activates macrophages and induces tissue damage. In the retina, S100B is expressed by astrocytes and Müller glia and can stimulate the expression of inflammatory cytokine by interacting with RAGE to activate PI3K/AKT, NF-κB, p38MAPK and JNK [26, 27]. Inhibition of the binding of the S100 protein family to RAGE could be a potential target for delaying disease progression in the early stages of DR.

**Fig. 2:**
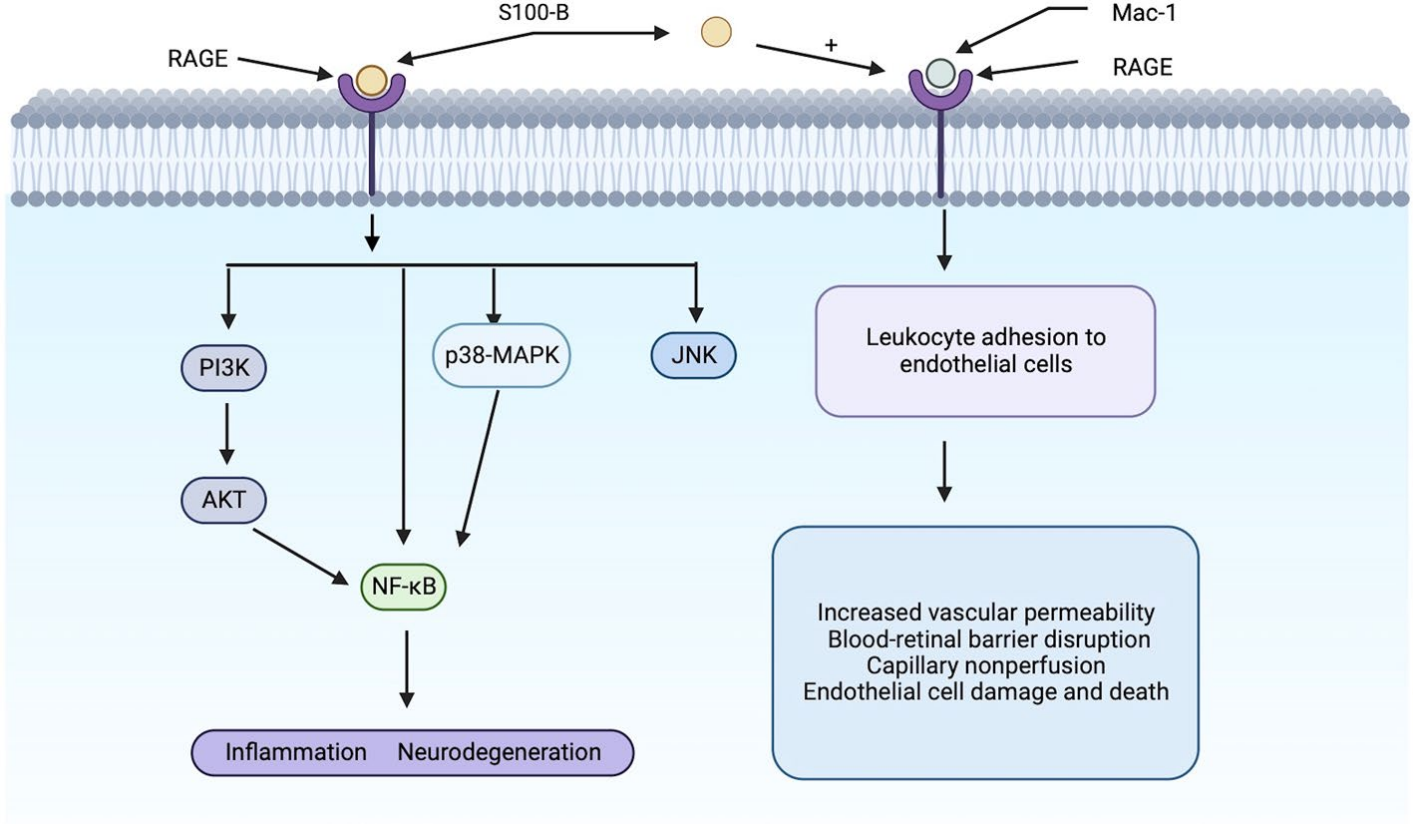
The S100 protein family interacts with RAGE can activate NF-κB, and S100-B enhances the binding of RAGE to Mac-1. Binding of RAGE to Mac-1 mediates leukocyte adhesion to endothelial cells

The core of DR early pathology is an increase in retinal ICAM-1 and neutrophil surface integrins. ICAM-1 is an important inflammatory adhesion molecule participated in the interaction between leukocyte and endothelial cells, increasing vascular permeability, leading to BRB rupture, capillary nonperfusion and endothelial cells damage and death [7]. RAGE can act as an endothelial adhesion receptor for leukocyte integrins and directly regulate leukocyte recruitment. The binding of RAGE to β2-integrin Mac-1 (CD11a/CD18) on leukocyte mediates leukocyte adhesion to endothelial cells, and S100-B enhances the binding of RAGE to Mac-1. The mechanism by which the RAGE-Mac-1 interaction mediates leukocyte recruitment is applicable to pathophysiological conditions related to high RAGE expression, such as diabetes, chronic immune response, atherosclerosis or cancer. Antagonizing the interaction between RAGE and β2-integrin Mac-1 may provide a novel therapeutic strategy for diabetes or chronic immune responses associated with high RAGE expression [28] (Fig. 2).

## RAGE is involved in promoting neovascularization

Activation of AGEs-RAGE signaling induces Müller cell activation and upregulates VEGF expression, leading to retinal vascular endothelial damage and promoting neovascularization [29].

PI3K/AKT signaling plays a key role in neovascularization in DR. Activation of PI3K/AKT signaling by AGEs combined with RAGE may prolong endothelial cell survival, and cooperate with VEGF to promote retinal endothelial cell proliferation and migration, ultimately promoting neovascularization [30, 31]. Activated AKT can mediate a variety of downstream target proteins, such as cell growth and proliferation through phosphorylation of mTOR, and apoptosis through inhibition of adverse expression [32, 33].

Li et al. found that RAGE interacts with AGEs to activate Src kinase, and the downstream signal transduction pathways of Src include PI3K, FAK and MAPK. Activated Src not only promotes angiogenesis through downstream signaling pathways, but also transmits signals to moesin, VE-cadherin and FAK by enhancing ERK phosphorylation in endothelial cells, causing damage to the endothelial cell barrier and increased vascular permeability [34].

Moesin is a connecting protein between actin microfilaments and the cell membranes, which represents the most important ERM protein in endothelial cells. Its phosphorylation can alter cell morphology, motility and polarity [35], promoting efficient serous membrane contraction during angiogenic endothelial cell migration [36–38].

**Fig. 3:**
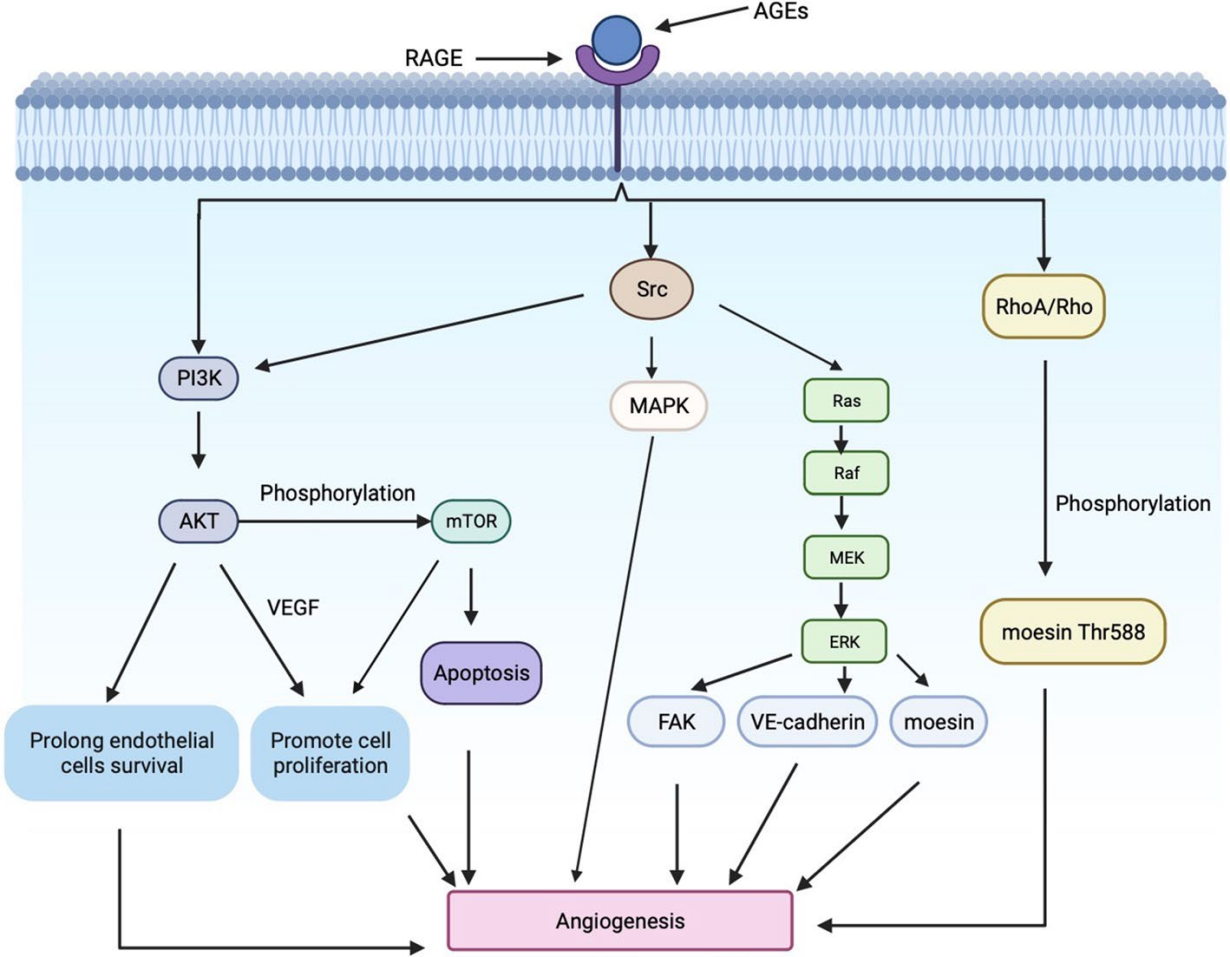
RAGE bind to AGEs can promote neovascularization

Chen et al. showed that RAGE binds to AGEs, activates the RhoA/RHO-associated protein kinase (ROCK) signaling pathway, and induces phosphorylation of threonine residue 558 (Thr558) of moesin. In this way, it promotes vascular endothelial cell contraction, vascular endothelial barrier disruption, increased vascular endothelial permeability, and ultimately angiogenesis. Thus the AGEs-RAGE axis and moesin may be candidate targets for overcoming diseases associated with pathologic neovascularization [39] (Fig. 3).

Glyceraldehyde-derived AGE (AGE2) and glycolaldehyde-derived AGE (AGE3) are toxic and can induce excessive angiogenesis, which plays an important role in the progression of DR. Yamazaki et al. found that AGE3 activated the mTOR signaling pathway in endothelial cells by binding to scavenger receptors (CD163, CD36 and LOX-1) and promoted angiogenesis excessively. RAGE helps toxic AGEs to promote angiogenesis by inducing increased expression of scavenger receptors [40].

## RAGE interacts with HMGB1 to promote the release of inflammatory factors and neovascularization

HMGB-1 is a highly conserved non-histone DNA-binding protein that stabilizes nucleosome formation and promotes transcription in the nucleus, and it is released both passively from necrotic cells and actively from monocytes/macrophages and endothelial cells. HMGB1 acts as both a pro-inflammatory cytokine and a pro-angiogenic factor. Pachydaki et al. were the first to report that the vitreous cavity and preretinal membranes of PDR patients had increased levels of RAGE and its ligand HMGB1 [41]. Abu El-Asrar et al. further demonstrated that HMGB1 levels in the vitreous of PDR patients were positively correlated with the severity of neovascularization [42–44]. The discovery of Shen suggests the presence of pro-inflammatory and angiogenic states in PDR, as evidenced by elevated levels of HMGB-1, RAGE, VEGF and IL-1β in vitreous and serum. HMGB-1 may be a cytokine that correlates the inflammatory response with angiogenesis in DR, and may be a new therapeutic target to inhibit the progression of PDR [6].

HMGB1/RAGE mediated signaling cascades include ERK1/2, p38 MAPK and NF-κB pathways. Activation of the HMGB1/RAGE axis and downstream pathways contributes to the pro-inflammatory and pro-angiogenic effects of retinal endothelial cells, which destroys the function of the retinal vascular barrier of the rats, and plays an important role in the pathological response to DR [45–47].

In terms of pro-inflammatory effects, HMGB-1 signals through RAGE to activate NF-κB, up-regulate the expression of pro-inflammatory cytokines, growth factors and chemokines, and increase the expression of ICAM-1 and VCAM-1 on the surface of endothelial cells. RAGE also contributes to the activation of Mac-1 on neutrophils. The interaction between Mac-1 and ICAM-1 reactivates NF-κB [45, 47]. Orlova et al. reported that HMGB1 dose-dependently enhanced the interaction between Mac-1 and RAGE and induced NF-κB activation in neutrophils [48].

As for angiogenesis, endothelial cells express HMGB1, as well as the receptors RAGE, TLR2 and TLR4. HMGB1 binds to RAGE to activate MAPK, including ERK1/2, p38MAPK and JNK, and ultimately the transcription factor NF-κB. HMGB1 interacts with TLR4 to activate IKK-β and IKK-α, facilitate nuclear translocation of activated NF-κB, increase the expression of pro-inflammatory factors and pro-angiogenic genes, and induce the release of a variety of pro-inflammatory and pro-angiogenic cytokines in hematopoietic cells and endothelial cells. All of these are involved in activating EC, macrophages, EPC, and mesoangioblasts fibroblastic factors, which contribute to the formation of blood vessels [49–52]. NF-κB can also promote apoptosis and inhibit cell proliferation. HMGB1 induces apoptosis of retinal vascular endothelial cells through the NF-κB pathway, creating a nonperfusion zone, which then induces compensatory proliferation and increased neovascularization of blood vessels [53]. The level of HMGB1 were elevated in patients with advanced DR. Similarly, diabetic patients have increased levels of RAGE and TLR4 polymorphisms. Studies of diabetic animal models and cells have shown that a key role of the HMGB1/TLR4/RAGE axis is to phosphorylate NF-κB, leading to the activation of a number of inflammatory cytokines, causing retinal damage and increased levels of retinal inflammatory mediators. In a word, HMGB1 signaling can activate downstream NF-κB by activating TLR4 and RAGE pathways, promoting retinal inflammation and angiogenesis in DR and exacerbating the progression of DR [54].

**Fig. 4:**
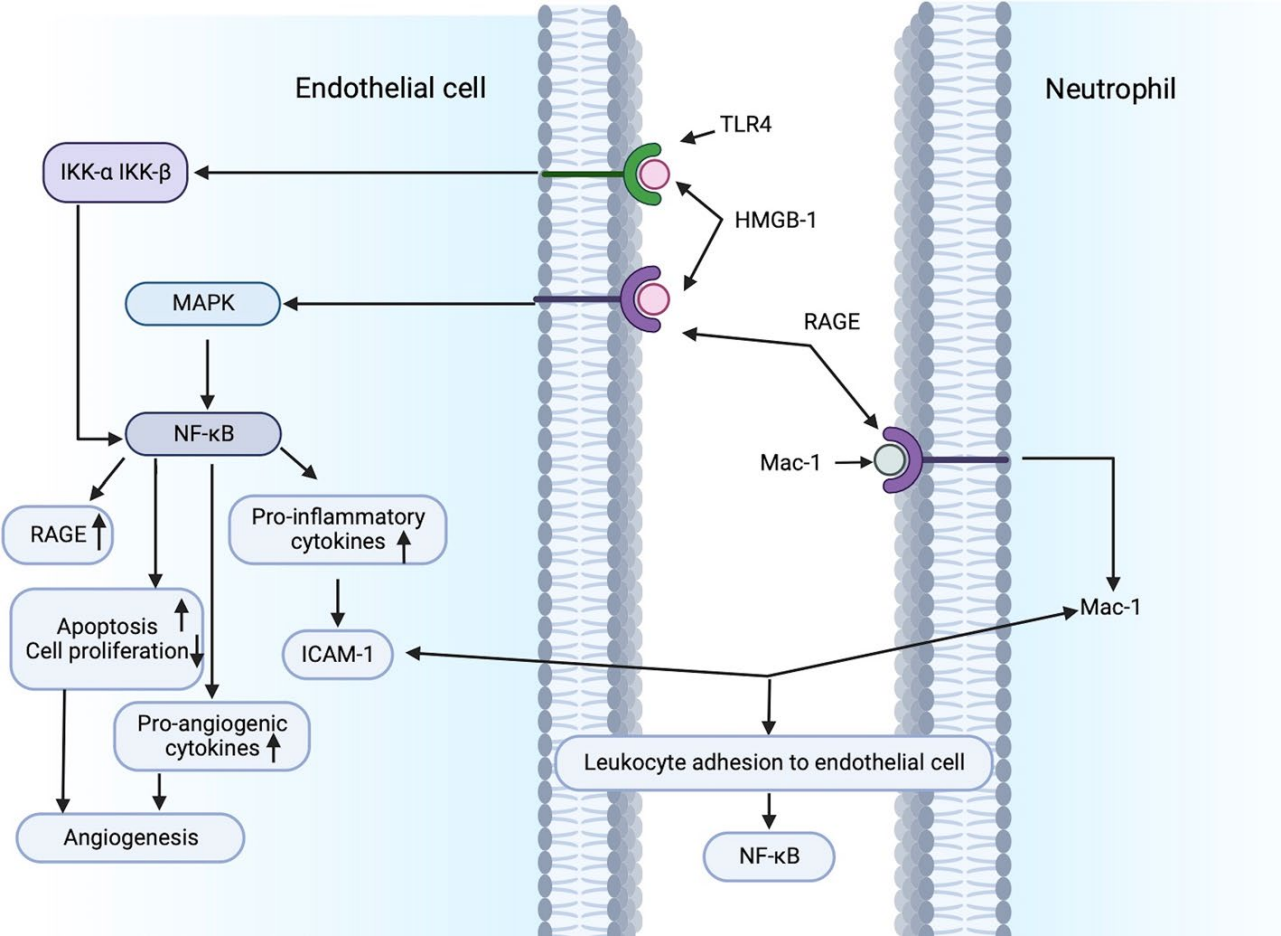
RAGE interacts with HMGB1 to promote inflammatory factors release and neovascularization. RAGE contributes to activate Mac-1 on neutrophils. Mac-1 and ICAM-1 combine to reactivate NF-κB

The existence of NF-κB binding sites in the RAGE promoter creates positive feedback by increasing RAGE expression. Thus, the cascade response activated by the interaction of HMGB1 with RAGE can be amplified by upregulating RAGE expression, contributing to initiate and maintain retinal inflammation and angiogenesis under pathological conditions [55] (Fig. 4).

Therefore, the data suggest that HMGB1/RAGE/TLR4 causes inflammatory damage and neovascularization of DR, and inhibiting its effects may become a new field for therapeutic development. RAGE inhibitors can block HMGB1-induced neovascularization in vivo. When cells were cultured in high glucose, the inhibition of RAGE reduced the inflammatory response [56]. The use of RAGE fusion proteins in streptozotocin-induced diabetic animal models demonstrated that inhibition of RAGE prevents diabetes-associated vascular permeability injury [54, 57].

The research on diabetic TLR4 knockout mice by Wang H and others showed that TLR4 can drive retinal inflammation, leading to increased levels of VEGF, TNFα and IL-β in the retina, indicating that TLR4 is critical for DR inflammation. In RGC grown in high glucose, inhibition of TLR4 reduced apoptosis and inflammatory factors [58–60]. Inhibition of TLR4 through multiple pathways may become a novel therapeutic approach for DR [54].

## Discussion

The importance of DR is further emphasized by the fact that it is one of the most common causes of blindness worldwide. DR is increasingly recognized as a neurovascular disease of the retina. Degenerative lesions of retinal nerves are accompanied by vascular changes, and changes in neuronal function can be detected clinically prior to the appearance of vascular diseases [17, 61]. Most treatments are now focused on the late-stage complications of DR and few have developed treatments for early lesions. DR can be treated and prevented further complications with laser photocoagulation, intravitreal injection of drugs, such as VEGF inhibitors and steroids and vitrectomy, but structural abnormalities, such as neurovascular damage that have already occurred in the retina cannot be reversed. Therefore, there is an urgent need for topical or systemic therapies based on the pathogenesis to prevent, stop, reverse or at least delay retinopathy at an early stage, and to save patients' vision as much as possible [4].

RAGE plays an important role in the pathogenesis of DR. RAGE binding to multiple ligands generates a series of cascade reactions by activating downstream signaling pathways, leading to NF-κB activation, gene transcription of inflammatory mediators, neurodegeneration and angiogenesis, which are critical in the pathogenesis of DR. Therefore, factors that target RAGE, interrupt RAGE−ligand interactions and inhibit activated downstream signaling pathways appear to be emerging research directions for the prevention and/or treatment of DR [62].

## Conclusion

There is growing evidence that RAGE can initiate and maintain significant cell perturbations in the inner and outer layers of the retina, and plays a key role in the pathogenesis of DR. Therefore, inhibition of RAGE or the downstream signaling pathways may be a valuable research direction for future treatment of DR.

More studies are needed to explore the mechanism of RAGE and its activated downstream signaling pathways in retinal inflammatory activation, neurodegeneration and neovascularization. This may identify potential therapeutic targets for DR, which will be important for the prevention of DR-related blindness. In addition, with regard to the long-term effects of RAGE-targeted therapies in humans, future investigations are needed to improve the understanding of the properties of RAGE inhibitors.

## Data Availability

This declaration is not applicable.
